# Effects of ligand distribution on receptor-diffusion-mediated cellular uptake of nanoparticles

**DOI:** 10.1098/rsos.170063

**Published:** 2017-05-31

**Authors:** Long Li, Yudie Zhang, Jizeng Wang

**Affiliations:** Key Laboratory of Mechanics on Disaster and Environment in Western China, Ministry of Education, College of Civil Engineering and Mechanics, Lanzhou University, Lanzhou, Gansu 730000, People's Republic of China

**Keywords:** cellular uptake, receptor diffusion, optimal ligand distribution, controlled-release drug delivery

## Abstract

Biophysical-factor-dependent cellular uptake of nanoparticles (NPs) through receptor-diffusion-mediated endocytosis bears significance in pathology, cellular immunity and drug-delivery systems. Advanced nanotechnology of NP synthesis provides methods for modifying NP surface with different ligand distributions. However, no report discusses effects of ligand distribution on NP surface on receptor-diffusion-mediated cellular uptake. In this article, we used a statistical dynamics model of receptor-diffusion-mediated endocytosis to examine ligand-distribution-dependent cellular uptake dynamics by considering that ligand–receptor complexes drive engulfing to overcome resistance to membrane deformation and changes in configuration entropy of receptors. Results showed that cellular internalization of NPs strongly depended on ligand distribution and that cellular-uptake efficiency of NPs was high when ligand distribution was within a range around uniform distribution. This feature of endocytosis ensures robust infection ability of viruses to enter host cells. Interestingly, results also indicated that optimal ligand distribution associated with highest cellular-uptake efficiency slightly depends on distribution pattern of ligands and density of receptors, and the optimal distribution becomes uniform when receptor density is sufficiently large. Position of initial contact point is also a factor affecting dynamic wrapping. This study explains why most enveloped viruses present almost homogeneous ligand distribution and is useful in designing controlled-release drug-delivery systems.

## 1. Introduction

Nanoparticle (NP) uptake into cells through receptor-mediated endocytosis is crucial in nanomedicine and virology [1–3]. In general, receptor–ligand complexes form when mobile receptors on cell membrane diffuse to binding sites (ligands) on NP surface. Binding of receptor–ligand complex can drive cellular uptake. NPs are treated as potential carriers in biomedical applications due to this characteristic in biological systems. Therefore, knowledge on NP–cell interactions can greatly contribute to fundamental biological understanding and practical applications, including nanotoxicology, pharmacology and drug delivery [4–9].

In the past decade, a number of studies were conducted to investigate influence of biophysical factors on cellular uptake of NPs. These studies successfully demonstrated that cellular uptake depends on size [10–13], shape [14–16], orientation [14,17–19], stiffness [20–22], cytoskeleton viscoelasticity [18,23–25], cooperativity [26,27], stochastic adhesion [28], membrane wrapping [29–33] and surface charge of NPs [34–37]. Most of these studies assumed that ligands are uniformly distributed on NP surface. However, reports rarely mentioned how ligand distribution affects internalization of NPs into cells.

Naturally, most enveloped viruses are typical NPs and must use cell processes to replicate, thus promoting dramatic biochemical and structural changes in host cells and eventually leading to cell death. Viruses should enter host cells through receptor–ligand affinity to enable effective infection of viral particles. Uniform ligand distribution on viral capsid is revealed through experimental advances. Glycoprotein ligands are fixed in envelope membranes, especially for human immunodeficiency viruses, and are homogeneously distributed on membranes of mature viruses through ultrastructural cytochemistry and morphometry [38]. One issue raised is whether uniform ligand distribution of enveloped virus guarantees its uptake into host cells. Advances in NP synthesis provide various approaches to modify ligand distribution [39–41]. However, no method can enable controlled drug release by ligand distribution in drug-delivery systems.

Recently, Schubertová *et al*. [42] performed extensive coarse-grained molecular dynamics simulations to explore effects of ligand distribution on rate of cellular uptake of NPs, where receptor diffusion is inhibited by setting extremely high density, and ligands on NPs are either diffusible or immobile. By considering various cases of different ligand distributions, the researchers discovered that NPs with homogeneous ligand distribution are most efficiently wrapped by cell membrane, as inhomogeneous distribution of ligands may increase activation energy and reduce uptake efficiency. Although this finding is interesting, in most cases, receptor diffusion plays an important role in NP uptake in normal cells [5,8–10,16,18,43]. For receptor-diffusion-mediated uptake of NPs, no study can clarify how distribution of ligands and diffusion of mobile receptors with low average density coupling influence cellular uptake of NPs.

In this article, we used statistical dynamics model of endocytosis [10,44] by considering cell membranes embedded with diffusive mobile receptors wrapped around cylindrical particles coated with differently distributed ligands to investigate effects of ligand distribution on cellular uptake.

## 2. Model

**Figure 1.**
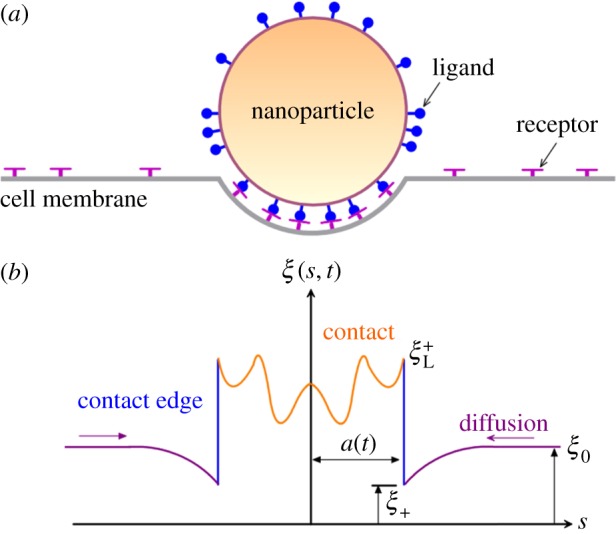
Schematic diagram of cellular uptake of NPs with different ligand distributions. (*a*) Remote mobile receptors diffusing to binding sites to drive cellular uptake. (*b*) Receptor density distribution along cell membrane. At the contact region, the receptor density is not constant.

Figure 1 displays cell membrane containing mobile receptor wrapped around a cylindrical NP of radius *R* and coated with immobile ligands. Different from previous dynamic models [10,18], ligand density *ξ*_L_ is no longer a constant but a function along arc length, *x*, of the cross section of NPs. For mobile receptors, we assumed an initially uniform density, *ξ*_0_. Once mobile receptors diffuse to binding sites and bind with ligands on particle surface, receptor density within the contact area becomes identical to ligand density *ξ*_L_ and may result in local depletion and lead receptor diffusion towards the contact zone, as shown in figure 1*b*. Contact edge (either left or right) is denoted as *s *= *a*(*t*).

For free receptor outside the contact region, for example, *s* ≥ *a *(*t*), continuity and diffusion equations on receptor density *ξ*(*s*, *t*) are given as follows [10,18,44]:

2.1 $$\frac{\partial \xi (s,t)}{\partial t}=-\frac{\partial j(s,t)}{\partial s}=D\frac{\partial^{2}\xi (s,t)}{\partial s^{2}},$$

where j(s,t)=−D(∂ξ(s,t)/∂s), that is, receptor diffusion flux with diffusion coefficient *D*.

Within contact region, *s *< *a*(*t*), *ξ*(*s*, *t*) = *ξ*_L_(*s*) and *j*(*s*, *t*) = 0. Conservation of membrane receptors can be obtained using the following:

2.2 $$\frac{\text{d}}{\text{d}t}\left[\int_{0}^{a(t)}\xi_{L}(s)\text{d}s+\int_{a(t)}^{\infty }\xi (s,t)\text{d}s\right]=0,$$

from which one can deduce the formula

2.3 $$(\xi_{L}^{+}-\xi_{+})\frac{\text{d}a(t)}{\text{d}t}+j_{+}=0\;\text{on}\;s=a(t),$$

where $\xi_{L}^{+}\equiv \xi_{L}(a^{+})$, $\xi_{+}\equiv \xi (a^{+},t)$ and $j_{+}\equiv j(a^{+},t)$ stand for receptor–ligand bond density, receptor density and flux in front of contact edge, respectively. We assumed that $\xi (s,t)\to \xi_{0}$ and j(s,t)→0 for s→∞.

Formation of ligand–receptor complexes drives engulfing to overcome resistance from membrane deformation and changes in configuration entropy of receptors during NP–cell contact. Hence, after normalization by *k*_B_*T* (*k*_B_ represents Boltzmann constant and *T* refers to absolute temperature), free energy function for cellular uptake of NPs can be derived using the equation [44,45]

2.4 F(t)=∫0a(t)(−ξL(s)eRL+ξL(s) ln ⁡ξL(s)ξ0+12Bκp2)ds+∫a(t)∞ξ(s,t) ln ⁡ξ(s,t)ξ0ds,

where *e*_RL_ refers to normalized adhesion energy of a receptor–ligand pair; *B* corresponds to normalized bending modulus; *κ*_p_=1/*R* represents stress-free curvature of membrane; and kBT ln ξL/ξ0 and kBT ln ⁡ξ/ξ0 are energy values per receptor associated with loss of configuration entropy of bonds and free receptors. We noted that a similar mathematical framework was developed in previous studies of biological membranes spreading on substrates coated with uniform [44] and non-uniform ligands [45].

Differentiating equation (2.4) with respect to time leads to the following formula:

2.5 dF(t)dt=−(ξL+eRL−12Bκp2−ξL+ ln ⁡ξL+ξ+ +ξL+−ξ+ )da(t)dt−∫a(t)∞Dξ(∂[ ln ⁡(ξ/ξ0)+1]∂s)2ds.

For a power-balanced process, decrease in rate of free energy should be equal to energy dissipated from receptor diffusion. Therefore, power balance equation features the form

2.6 ξL+eRL−12Bκp2−ξL+ ln ⁡ξL+ξ++ξL+−ξ+=0.

For a given ligand density *ξ*_L_, diffusion equation (2.1) for receptor density should be subjected to boundary conditions $\xi (\infty ,t)\to \xi_{0}$ and $\xi_{+}\equiv \xi (a^{+},t)$ provided by equation (2.6). No simple analytical solution for equation (2.1) exists for arbitrary ligand density in contrast with uniform ligand distribution [10]. Equation (2.1) can be calculated numerically through adopting finite difference method (for a detailed description of the method, see our previous study [18]). We assumed independent wrapping at two sides of initial contact point, and they can be determined by equations (2.1)–(2.6). Once the sum of two contact edges reaches 2*πR*, cellular uptake is completed and wrapping time is determined.

## 3. Results and discussions

**Figure 2.**
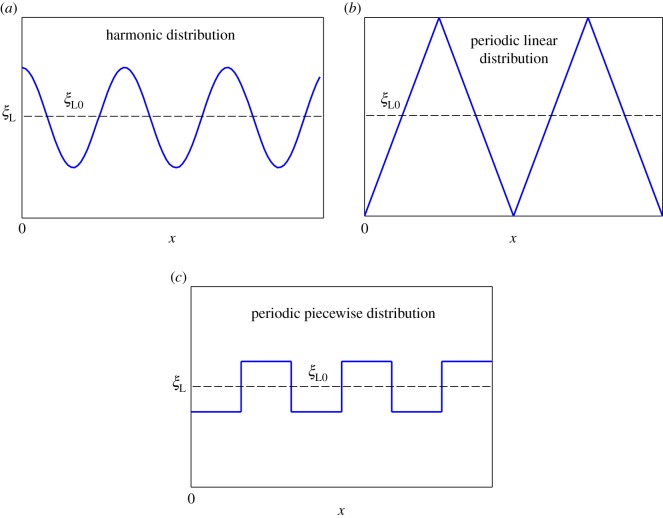
Different ligand distributions considered in the current work. (*a*) Harmonic distribution, (*b*) periodic linear distribution and (*c*) periodic piecewise distribution.

Hereafter, we used typical values of particle radius *R *= 20 nm, binding energy of single bond *e*_RL_* *= 15 [46], bending modulus of cell membrane *B *= 20 [47], diffusion coefficient of receptors on membrane *D *= 10^4^ nm^2^ s^–1^ [46] and initial receptor densities *ξ*_0_* *= 50, [10] 500 µm^–2^ [10]. By fixing average ligand distribution as *ξ*_L0_, we considered the three types of ligand distributions, as shown in figure 2:

Case A. Harmonic distribution

ξL(x)=ξL0[1+A sin (2λxR+π2)]forx≤2πR;

Case B. Periodic linear distribution

$$\xi_{L}(x)=\left\{ \begin{array}{ll} \frac{4n\xi_{L0}}{T_{l}}x+\xi_{L0}(1-n), & 0\leq x\leq 0.5T_{l}\\ -\frac{4n\xi_{L0}}{T_{l}}x+\xi_{L0}(1+3n), & 0.5T_{l}<x\leq T_{l} \end{array}\right.\;\text{for}\;x\leq 2\pi R;\;\text{and}$$

Case C. Periodic piecewise distribution

$$\xi_{L}(x)=\left\{ \begin{array}{ll} \xi_{L0}-m\xi_{L0}, & 0<x<0.5T_{p}\\ \xi_{L0}, & x=0,0.5T_{p},T_{p}\\ \xi_{L0}+m\xi_{L0}, & 0.5T_{p}<x<T_{p} \end{array}\right.\;\text{for}\;x\leq 2\pi R,$$

where *ξ*_L0_* *= 5000 µm^–2^ [18], that is, average ligand density; *Aξ*_L0_ and 2*λ*/*R* represent amplitude and frequency of ligand density for harmonic distribution, respectively; *λ* corresponds to positive whole numbers; *ξ*_L0_(1 + *n*) and *ξ*_L0_(1 − *n*) refer to maximum and minimum ligand density in linear distribution, respectively; *T*_l_= 2*πR*/*N*_l_ denotes period of ligand density with periodic linear distribution; *N*_l_ is cycle number; and *T*_p_= 2*πR*/*N*_p_ and *mξ*_L0_ stand for period and amplitude for ligand density under periodic piecewise distribution with cycle number *N*_p_, respectively.

**Figure 3.**
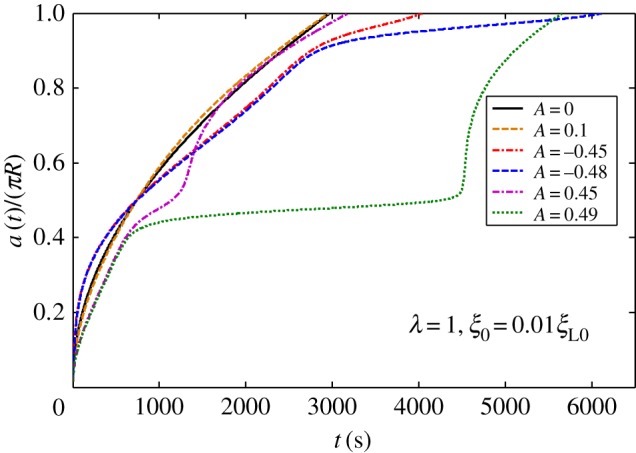
Normalized wrapping degree as a function of time for different amplitudes of ligand density with *λ *= 1 and initial receptor density *ξ*_0_* *= 0.01*ξ*_L0_ in harmonic ligand distribution.

**Figure 4.**
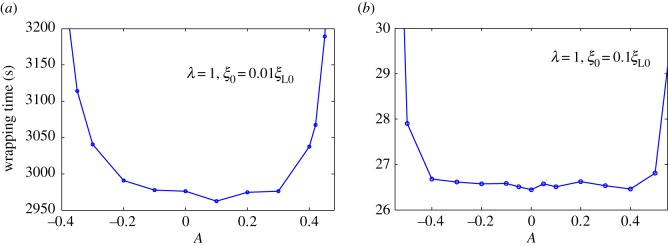
Wrapping time as a function of amplitude of ligand density for *λ *= 1, and initial receptor density at (*a*) *ξ*_0_* *= 0.01*ξ*_L0_ and (*b*) *ξ*_0_* *= 0.1*ξ*_L0_ in harmonic ligand distribution.

**Figure 5.**
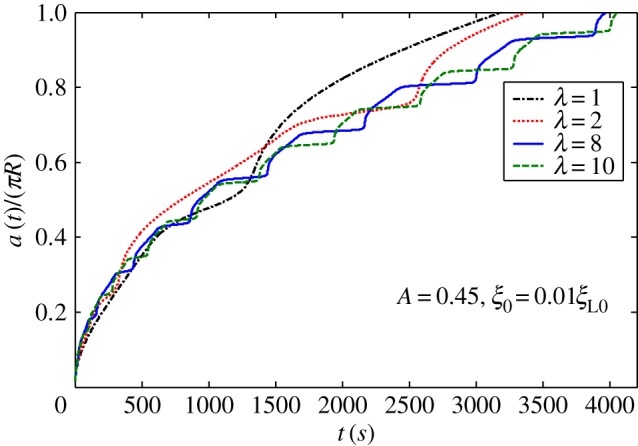
Normalized wrapping degree as a function of time for different frequencies of ligand density with *A *= 0.45 and initial receptor density *ξ*_0_* *= 0.01*ξ*_L0_ in harmonic ligand distribution.

Figure 2*a* shows harmonic distribution of ligands. Figure 3 plots numerically determined normalized wrapping degree, *a*/(*πR*), as a function of time at different amplitudes of ligand density with *λ *= 1 and position of initial contact point at *x *= 0. Figure 3 shows that ligand distribution can significantly influence uptake, and fastest cellular uptake associated with short wrapping time occurs when ligand density is close to *A *= 0.1. Figure 4 displays predicted relationships between wavy amplitude and wrapping time. This figure also indicates that optimal ligand distributions corresponding to a small wavy amplitude for low receptor density, 0.01*ξ*_L0_, and zero wavy amplitude for high receptor density, 0.1*ξ*_L0_, exist for the shortest wrapping time. Large wavy amplitude can lead to long wrapping time. Figure 5 presents influence of wavy frequency of ligands on dynamic wrapping. The figure also shows that at large frequencies, final wrapping time is almost independent of distribution frequency of ligands. For large wavy frequencies, aside from final wrapping times, whole wrapping procedures are almost identical.

**Figure 6.**
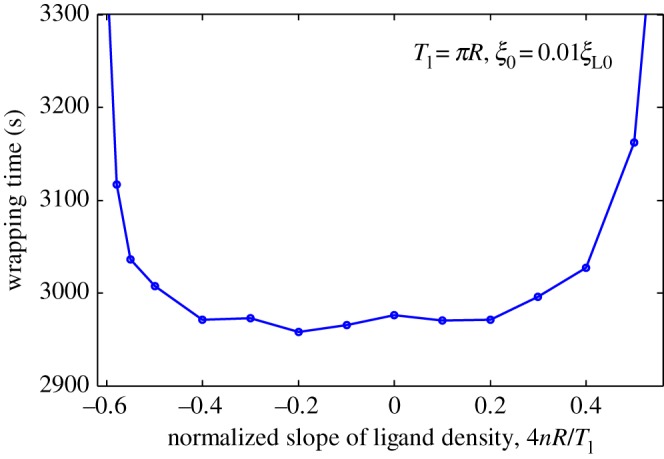
Wrapping time as a function of normalized slope of periodic linear-dependent ligand density for *T*_l_* *= *πR* and initial receptor density *ξ*_0_* *= 0.01*ξ*_L0_.

**Figure 7.**
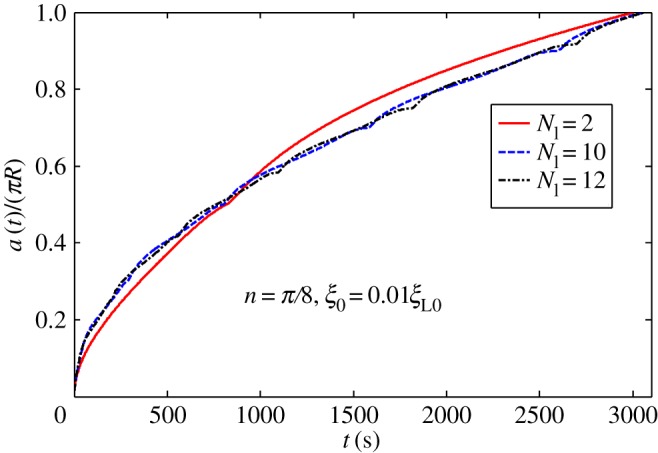
Normalized wrapping degree as a function of time for different cycle numbers of ligand density with *n *= *π*/8 and initial receptor density *ξ*_0_* *= 0.01*ξ*_L0_ in periodic linear ligand distribution.

Figure 2*b* shows periodic linear distribution of ligands. Figure 6 plots wrapping time as a function of normalized distribution slope 4*nR*/*T*_l_ when position of initial contact point is at *x *= 0. As indicated in figure 6, wrapping processes slightly differ at slope range of –0.4 to 0.4. When absolute value of slope becomes larger than 0.6, completion of wrapping becomes difficult. Figure 7 plots dynamic wrapping for different frequencies of ligand distribution and shows frequency of ligands that slightly affect the whole dynamic wrapping for periodic linear distribution of ligands.

**Figure 8.**
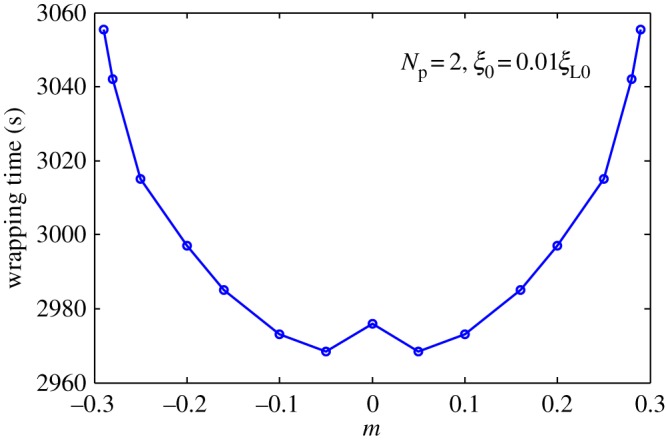
Wrapping time as a function of amplitude for cycle number *N*_p_* *= 2 and initial receptor density *ξ*_0_* *= 0.01*ξ*_L0_ in periodic piecewise distribution of ligand density.

**Figure 9.**
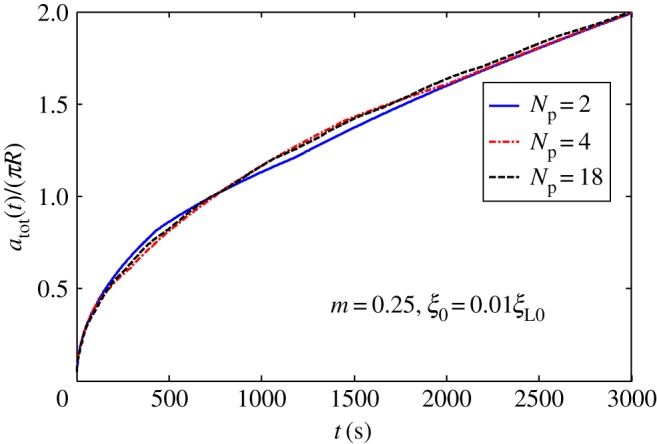
Normalized wrapping degree as a function of time for different cycle numbers for *m *= 0.25 and initial receptor density *ξ*_0_* *= 0.01*ξ*_L0_ in periodic piecewise distribution of ligand density.

In periodic piecewise, distribution of ligands shown in figure 2*c*. Figure 8 presents wrapping time as a function of wavy amplitude for cycle number *N*_p_* *= 2. Optimal ligand distribution for fastest uptake corresponds to a slightly wavy uniform distribution (|*m*|=0.05). Figure 9 depicts the relationship between normalized wrapping degree and time for different cycle numbers. Similar to effects of wavy frequency of ligand distribution to harmonic distribution and periodic linear distribution, only intermediate dynamic wrapping slightly depends on cycle number. Cycle number poses almost no effect on final wrapping time.

Based on the above discussions, slightly wavy pattern commonly results from three different cases of ligand distribution; this distribution corresponds to high uptake efficiency. Distribution is non-uniform in terms of wavy amplitude, which usually leads to increased wrapping time. This optimal distribution results from competition between driving force due to ligand–receptor binding and resistance from changes in configuration entropy of receptors. Interactions among NPs, non-uniform ligand distribution and cell membrane bending inevitably form local sparse or dense ligand–receptor bond densities at the contact area. For low ligand–receptor bond density, shortage of binding energy enlarges wrapping time. When local ligand–receptor density is high, wrapping time is also increased by high energy dissipated from changes in configuration entropy of receptors.

Bio-inspired methods from viruses suit designing of drug-delivery systems. Thus, NP–cell interactions must be biophysically understood. In figures 4, 6 and 8, fast wrapping exists in large-range ligand distribution around uniform distribution, providing physical insight into robust viral infection rather than gene expression [48,49]. From the perspective of physical optimization, optimal size (tens of nanometres) [10,11] and shape (sphere) [16] are revealed. In this study, we confirmed that ligand distribution is another significant factor determining receptor-diffusion-mediated NP uptake of cells. Almost uniform ligand distribution of viruses is possibly controlled by physical evolution and guarantees viral infectivity through receptor-mediated endocytosis.

Contrary to virus entry to host cells, NP capsules are sometimes expected to dissolve drugs over time, releasing these medical compounds at slower and steadier pace into target sites [50,51]. Therefore, according to the present study, controlled-release drug-delivery systems may be integrated by modifying ligand distribution on NP surface.

**Figure 10.**
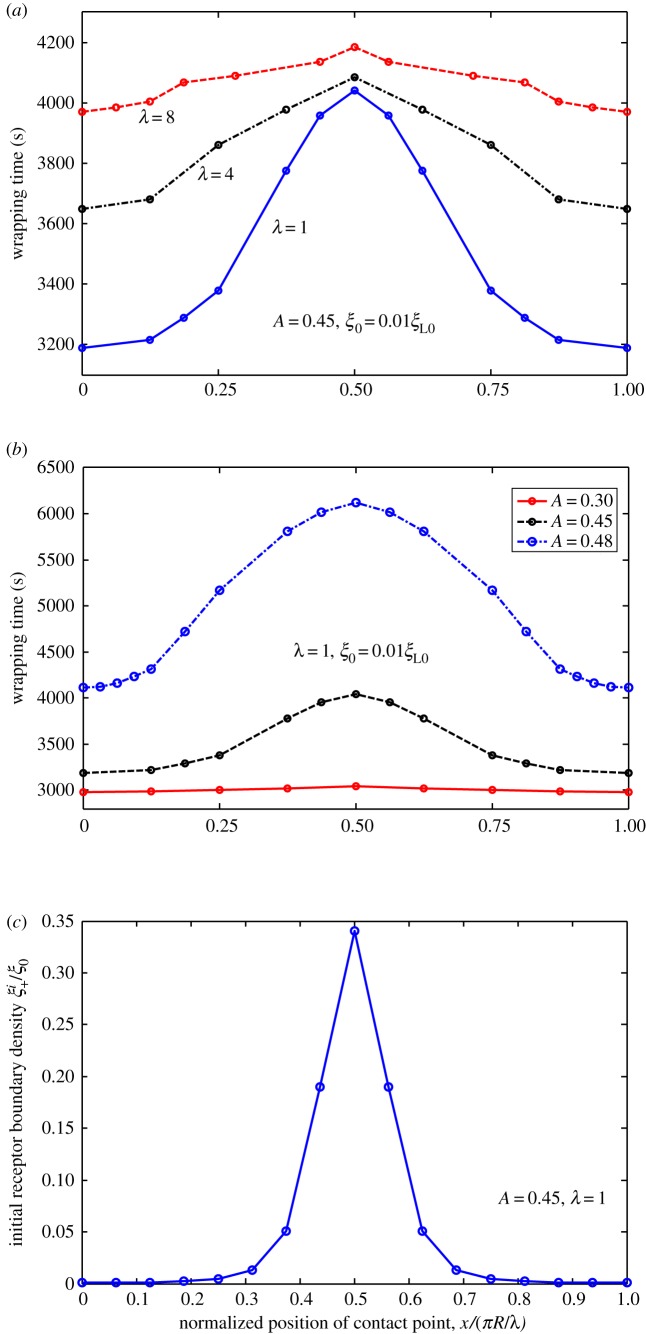
Wrapping time as a function of normalized position of contact point under different (*a*) frequencies and (*b*) amplitudes of ligand density for *ξ*_0_* *= 0.01*ξ*_L0_. (*c*) Variation in initial receptor boundary densities with normalized position of contact point for *λ *= 1. Harmonically distributed ligand density.

**Figure 11.**
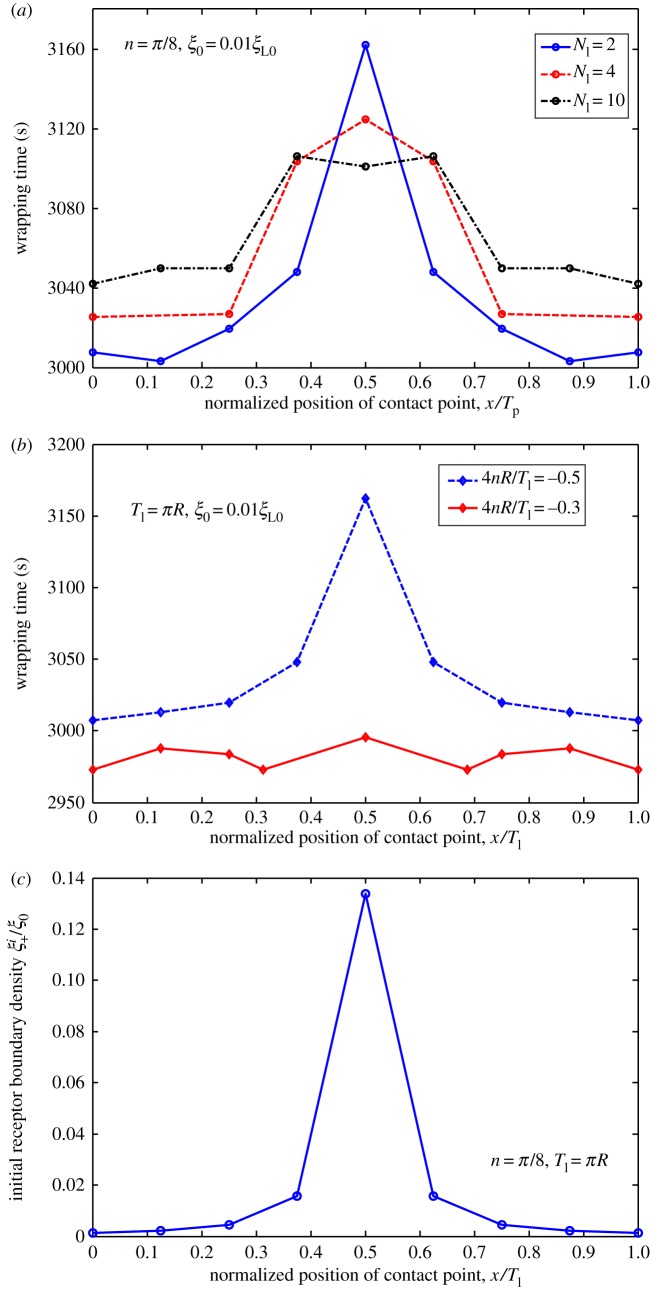
Wrapping time as a function of normalized positions of contact points under different (*a*) frequencies and (*b*) normalized slope of ligand density for *ξ*_0_* *= 0.01*ξ*_L0_. (*c*) Variation in initial receptor boundary density with normalized position of contact point for *n *= *π*/8, and *T*_l_* *= *πR*. Ligands with periodic linear distribution.

**Figure 12.**
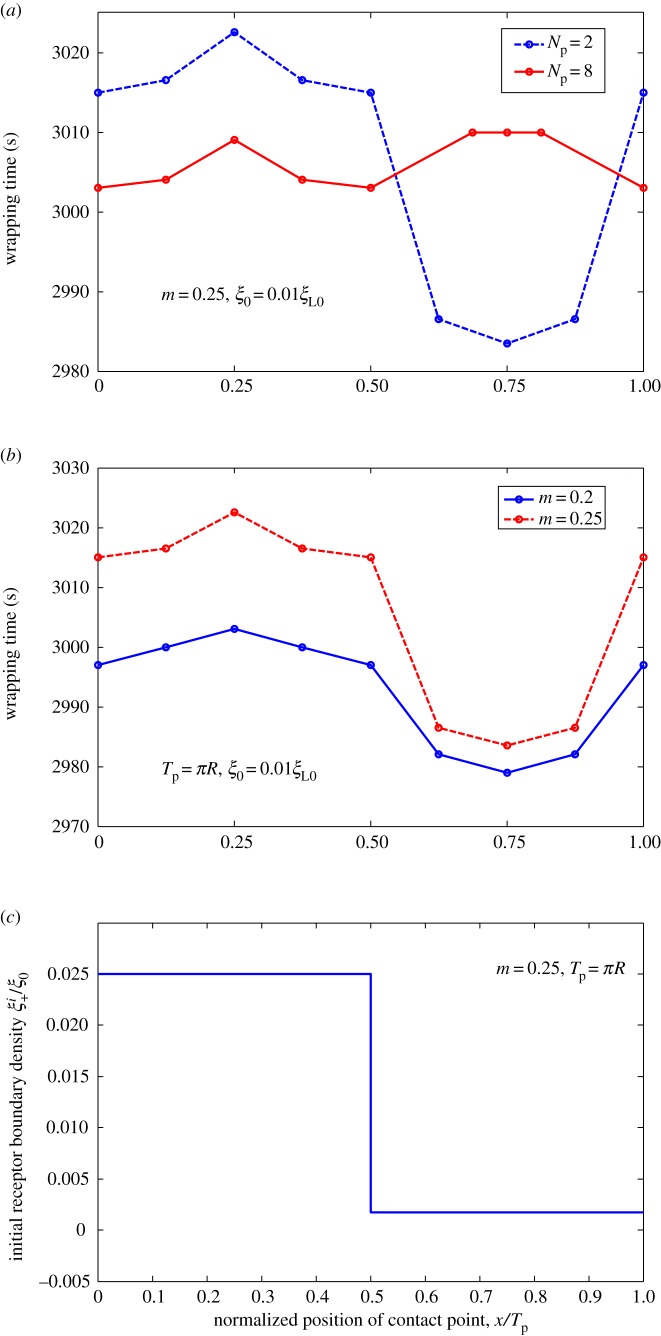
Wrapping time as a function of normalized positions of contact points under different (*a*) frequencies and (*b*) amplitude of ligand density for *ξ*_0_* *= 0.01*ξ*_L0_. (*c*) Variation in initial receptor boundary density with normalized positions of contact points for *m *= 0.25, and *T*_p_* *= *πR*. Ligands with periodic piecewise distribution.

We determined influence of ligand distribution characterized by wavy amplitude and frequency on cellular uptake of NPs. We hypothesized that slight difference in initial contact position of NP with fixed ligand distribution may lead to notable changes in total wrapping time, because wrapping is highly nonlinear. To justify this hypothesis, we considered uptake of NPs with different initial contact positions for cases of harmonic, periodic linear and periodic piecewise distributions. Wrapping time is numerically determined and plotted in figures 10–12 as a function of normalized positions of contact points under different amplitudes and frequencies of ligand distributions. Remarkably, dynamic cellular uptake can be significantly influenced by position changes in initial contact point when distribution amplitude is large, or when distribution frequency is low. By contrast, influence of positions of contact point on uptake decreases when wavy amplitude is low, or when wavy frequency is large.

Figures 10*c*, 11*c* and 12*c* show normalized initial boundary density of receptors as a function of normalized positions of initial contact points in harmonic, periodic linear and periodic piecewise distributions of ligands, respectively. Figures 10–12 show association of fastest wrapping with contact position of highest ligand density. Highest ligand density at initial contact position also corresponds to the largest gradient of initial receptor distribution, largest initial receptor diffusion flux and fastest initial wrapping speed. Hence, contact-point-position-dependent wrapping may be dependent on initial wrapping speed. A similar mechanism was revealed in our previous study [18] on cellular uptake of cylindrical NPs with different orientations.

Interestingly, Schubertová *et al*. [42] revealed a similar conclusion on uniform ligand distribution, which is most favorable for NP uptake, by performing coarse-grained molecular dynamics simulations in extreme cases, wherein receptors are ‘immobile' due to large density. In this study, figure 4 shows that uniform distribution features the most efficient case for NP uptake once average receptor density increases from 0.01*ξ*_L0_ to 0.1*ξ*_L0_. This finding is very similar to the case of ‘immobile' receptors studied by Schubertová *et al*. [42]. By contrast, optimal distribution of ligands is no longer uniform but becomes slightly wavy with amplitude near zero. Although Schubertová *et al*. [42] did not consider the effect of receptor diffusion at low density, they found that taking account of the effect of ligand diffusion will not change the fact that uniform distribution of ligands corresponds to the fastest uptake. We should note that the effect of ligand diffusion on the NP uptake is quite different from that of receptor diffusion. For example, in the case of very low receptor density, the NP still can be completely wrapped in as long as the density of ligands is sufficiently large; receptor diffusion causes this to happen and influences the total wrapping time. On the other hand, in the case of very low ligand density, no matter the density of receptor to be low or high, diffusion of ligands cannot make the NP to be wrapped in. When both the density of ligands and receptors are high, diffusion of either ligands or receptors becomes not important at all [42].

We aimed to determine how slightly non-uniform distribution of ligands correspond to the most efficient wrapping in cases of low-density receptors. Related mechanism involves long-time diffusion of NP to recruit enough receptors to binding sites under low receptor density. During this period, optimized local balance between adhesion energy as driving force and configurational entropy changes in diffusible receptors and membrane bending as resistance may change over time, resulting in spatial distribution pattern of ligands.

## 4. Conclusion

Based on the effect of NP ligand distribution on cellular uptake, we used statistical dynamics model of endocytosis by considering receptor–ligand binding, receptor diffusion and membrane deformation. We discovered that NP dynamic wrapping may depend on wavy-form ligand distribution and receptor density. We also discovered that wavy frequency almost features no effect on wrapping efficiency. By contrast, wavy amplitude significantly affects wrapping. The most efficient wrapping case corresponds to dependence of a range of slightly wavy forms of ligand distribution on receptor density. When receptor density is adequately high, optimized distribution of ligands becomes uniform. Slight variance in distribution almost does not change optimal states. As initial wrapping speed varies with positions of initial contact point, different positions of contact point between NP–cell membranes significantly affect wrapping when ligand distribution is in wavy form with large amplitudes and/or low frequency. These results provide physical understanding from an evolutionary view as to why enveloped viruses manifest almost homogeneous ligand distributions. Results indicate bio-inspired method of effective NP design for drug-delivery systems.

This study is restricted by several limitations. Cylindrical NPs and membrane surface tension are not considered. Kinetic reaction between receptor and ligand molecules [32,46,52–54], and viscoelastic deformation of cytoskeleton [23,55] are neglected.

## Data accessibility

All data are included within the article.
